# Molecular Detection and Phylogenetic Analysis of Orf Virus From Dermatological Lesions in the Teats of Goats

**DOI:** 10.1002/vms3.70139

**Published:** 2025-01-10

**Authors:** Yakup Yıldırım, Seval Bilge Dağalp, Gökhan Bozkurt, Fırat Doğan, Ali Küçük

**Affiliations:** ^1^ Department of Virology, Faculty of Veterinary Medicine Burdur Mehmet Akif Ersoy University Burdur Türkiye; ^2^ Department of Virology, Faculty of Veterinary Medicine Ankara University Ankara Türkiye; ^3^ Department of Obstetrics & Gynecology, Faculty of Veterinary Medicine Burdur Mehmet Akif Ersoy University Burdur Türkiye; ^4^ Department of Virology, Faculty of Veterinary Medicine Hatay Mustafa Kemal University Burdur Türkiye

**Keywords:** goat, orf virus, phylogenetic analyses, semi‐nested PCR

## Abstract

**Background:**

The orf virus (ORFV) is a viral pathogen that primarily causes contagious ecthyma in humans and different ruminants. The infection, which is common worldwide, causes large‐scale economic losses to animal breeders.

**Objective and Methods:**

In this study, tissue samples collected from eight randomly selected goats with dermatological lesions on the teats were examined in different goat herds. B2L gene‐specific primer pairs (PP1, PP3 and PP4) were used to reveal the presence of ORFV by molecular methods and for phylogenetic analysis.

**Results:**

Viral DNA was detected in four of eight tissues using the semi‐nested PCR method. In addition, the data obtained by performing sequence analyses of the amplicons with positive results were compared with the information of different ORFV isolates registered in the GenBank database. Based on the sequence analysis of the field isolates obtained in our study, it was found that the nucleotide similarities among these isolates and those from Asian countries were 100%. Furthermore, ORFV isolates collected from different species and produced in Türkiye over various periods exhibited homologous nucleotide sequences with similarities ranging from 98.1% to 98.8%. In the phylogenetic tree drawn based on the B2L genomic region, it was observed that our field isolates were classified in Group I together with other Turkish and Asian strains.

**Conclusion:**

As a result, while other pathogenic agents are considered the cause of disease in goats with dermatological lesions on their mammary tissue, the ORFV should also be evaluated, and protection and control programs should be prepared accordingly.

## Graphical abstract

Viral DNA (orf virus) was detected in tissue samples using the semi‐nested PCR method. In addition, the data obtained by performing sequence analyses of the amplicons with positive results were compared with the information of different orf virus isolates registered in the GenBank database. Based on the sequence analysis of the field isolates obtained in our study, it was found that the nucleotide similarities among these isolates and those from Asian countries were 100%. Furthermore, orf virus isolates collected from different species and produced in Türkiye over various periods exhibited homologous nucleotide sequences with similarities ranging from 98.1% to 98.8%.

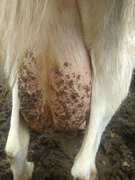

## 1 Introduction

Orf virus (ORFV) is a viral pathogen that mostly causes local dermatological infections in small ruminants, less frequently in different animal species and humans (Nandi, De, and Chowdhury 2011). The infection, which is common all over the world, causes economic losses, especially for sheep and goat breeders (MacLachlan et al. 2017). Taxonomically, the causative agent, ORFV, along with bovine pustular stomatitis virus, pseudocowpox virus and red deer poxvirus, which are in the *Chordopoxvirinae* subfamily of the *Poxviridae* family, have been classified in the *parapoxvirus* genus (Lefkowitz et al. 2018). *Parapoxviruses* are genetically and antigenically closely related, and their genome organization, virulence mechanisms, and morphologies are very similar (Karki et al. 2019; Spyrou and Valiakos 2015). The ORFV genome consists of linear double‐stranded DNA about 138–140 kb long with high G + C content. Like other poxviruses, the viral genome consists of a core region and variable genomic termini. Many gene regions with different functions have been identified in the structure of the virus (B2L, F1L, VIR, ORF109, GIF, ORF125, Vil‐10) (Li et al. 2023). The ORFV, which replicates in the cell cytoplasm, contains 132 open reading frames (ORFs), 88 of which are conserved across other chordopoxviruses. The central regions of the genome, particularly from ORF009 to ORF111, harbour functional genes involved in viral replication, maturation, and morphogenesis (Büttner and Rziha 2002; Mercer et al. 2002; Mercer et al. 2006). In the studies conducted, phylogenetic analyses of the B2L gene have identified three main genogroups of ORFVs (Kumar et al. 2014). The structural B2L gene with well‐conserved immunological features is frequently used in immunological and molecular studies related to ORFV (Karakas et al. 2013; Koç 2022; Spyrou and Valiakos 2015).

ORFV causes contagious pustular stomatitis and contagious ecthyma with dermatological lesions in small ruminants. ORFV forms highly contagious vesicula‐ulcerative pustules on keratinized skin and mucosal surfaces (Haig and Mercer 1998). Although many methods such as ELISA, virus isolation and electron microscopy are used for virus detection, qPCR, semi‐nested PCR and PCR techniques, which have higher sensitivity than others, are the most frequently used diagnostic methods (Bora et al. 2011; Nandi, De, and Chowdhury 2011).

This study aimed to investigate the role of ORFV in the aetiology of dermatological and pathological lesions such as papules, pustules and vesicles observed in the mammary tissues of goats in the sampled herds by molecular methods and to compare the obtained field viruses with their counterparts in the phylogenetic tree.

## 2 Materials and Methods

### 2.1 Sampling Animals and Clinical Samples

This study included 8 hair goats from 3 different herds, each consisting of 120–150 individuals in Burdur province in the west Mediterranean region of Türkiye. These goats exhibited dermatological lesions on the mammary tissue. Deformed mammary skin lesions were randomly collected from the 8 goats (3–4 years old). The goats were manually milked, and their lactation periods ranged from 60 to 90 days. Clinical findings observed on the goat mammary tissue included extensive papules and pustules. No signs of disease were observed in the mouth and lip areas. Furthermore, based on anamnesis, the animal owners and milkers did not exhibit any symptoms related to ORFV infection. The sampled animals had not been vaccinated against *parapoxvirus*, and no topical or systemic treatment was administered for mammary lesions.

After the samples were homogenized by scalpel in phosphate‐buffered saline with antibiotics (streptomycin and penicillin), they were stored at −80°C until testing.

A clinical examination of the goats was performed to assess local pain, redness, increased temperature and gangrenization of the mammary tissue, considering the possibility of mastitis due to skin lesions. The California Mastitis Test was conducted on milk samples collected from all animals. In addition, the visual appearance of the milk was examined.

### 2.2 PCR, Sequencing and Phylogenetic Analysis

DNA extraction from the skin lesion samples was performed according to Sambrook and Russell (2001). The semi‐nested PCR technique was carried out on DNA from all samples using the PP1, PP4 and PP3 oligonucleotide primers, which amplify sequences within the B2L gene encoding the major envelope protein. The sequences of PPP‐1, PPP‐4 (1134‐bp) and PPP‐3 (594‐bp) were 5′‐gtc gtc cac gat gag cag ct‐3′, 5′‐tac gtg gga agc gcc tcg ct‐3′ and 5′‐gcg agt ccg aga aga ata cg‐3′, respectively. These oligonucleotide primers are widely used for molecular identification and phylogenetic analysis of the ORFV (*parapoxvirus*) (Inoshima, Morooka, and Sentsui 2000). The PCR reactions were performed in two steps. For the first step, 3 µL (50 ng) of DNA was subjected to thermocycling in a 30‐µL reaction mixture containing 2.5 U of Taq DNA Polymerase (Fermentas, Lithuania), 3.5 mM dNTP mix (Fermentas, Lithuania), 10 pmol of each oligonucleotide primers (PP1 and PP4), 1.5 mM MgCl_2_ and 10X PCR buffer. The thermal cycling conditions were as follows: initial denaturation at 95°C for 5 min, followed by 35 cycles of denaturation at 94°C for 1 min, annealing at 55°C for 1 min and extension at 72°C for 1 min. A final extension step was performed at 72°C for 10 min. Semi‐nested PCR was then performed using 3 µL of the first PCR product with the PPP‐3 and PPP‐4 oligonucleotide primers under the same conditions. The PCR products were visualized on a transilluminator after electrophoresis in a 1% agarose gel containing Safe‐Red DNA stain (SafeViewTM Cat No. G108‐R, Canada). Approximately 25 mL of amplicon remaining from samples detected positive by gel electrophoresis was used for sequence analysis.

The PCR products were sequenced by a commercial company (Medsantek, Ankara) automatic sequence analyser (CEQ 8000; Beckman Coulter, Brea, CA, USA). Sequence assembly and editing were performed using the MUSCLE algorithm as implemented in Aliview Software before comparison with the GenBank nucleotide sequence database for sequence similarities using the Basic Length Alignment Search Tool (BLAST) software of the National Center for Biotechnology Information (NCBI) (Altschul et al. 1997; Larsson 2014). Phylogenetic analysis was performed by the neighbour‐joining method using MEGA X software (Kumar et al. 2018), based on the evolutionary distances between different sequences calculated using the Kimura two‐parameter model. The confidence level of the NJ tree was assessed by bootstrapping, using 1000 replicates. The nucleotide identities were calculated by using Matgat software. The sequences obtained were recorded in the GenBank database, and the accession number was obtained.

## 3 Results

The four samples were amplified using the PP3 and PP4 oligonucleotide primers. All positive samples were obtained from the same herd (Herd 2). Sequence analysis was performed on the field isolates to assess their genetic similarities with other ORFV isolates identified both globally and within Türkiye.

**FIGURE 1:**
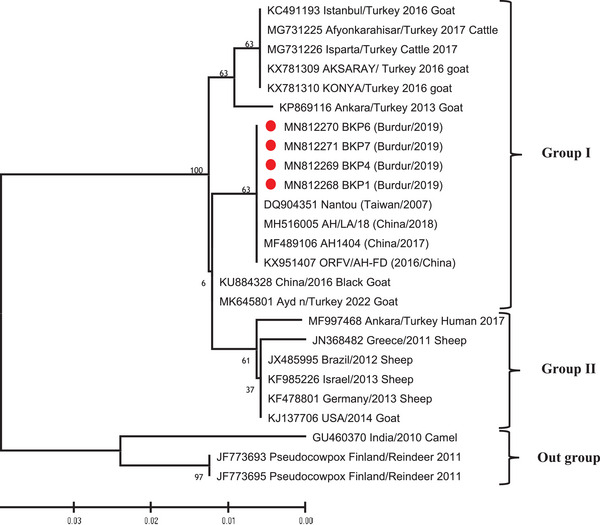
A rooted phylogenetic tree analysis of orf virus (ORFV). Neighbour‐joining tree calculated on partial B2L gene nucleotide sequences of orf viruses. The newly obtained sequences are marked with red circles. The scale bar shows the distance corresponding to 0.5 substitutions per nucleotide position.

**FIGURE 2:**
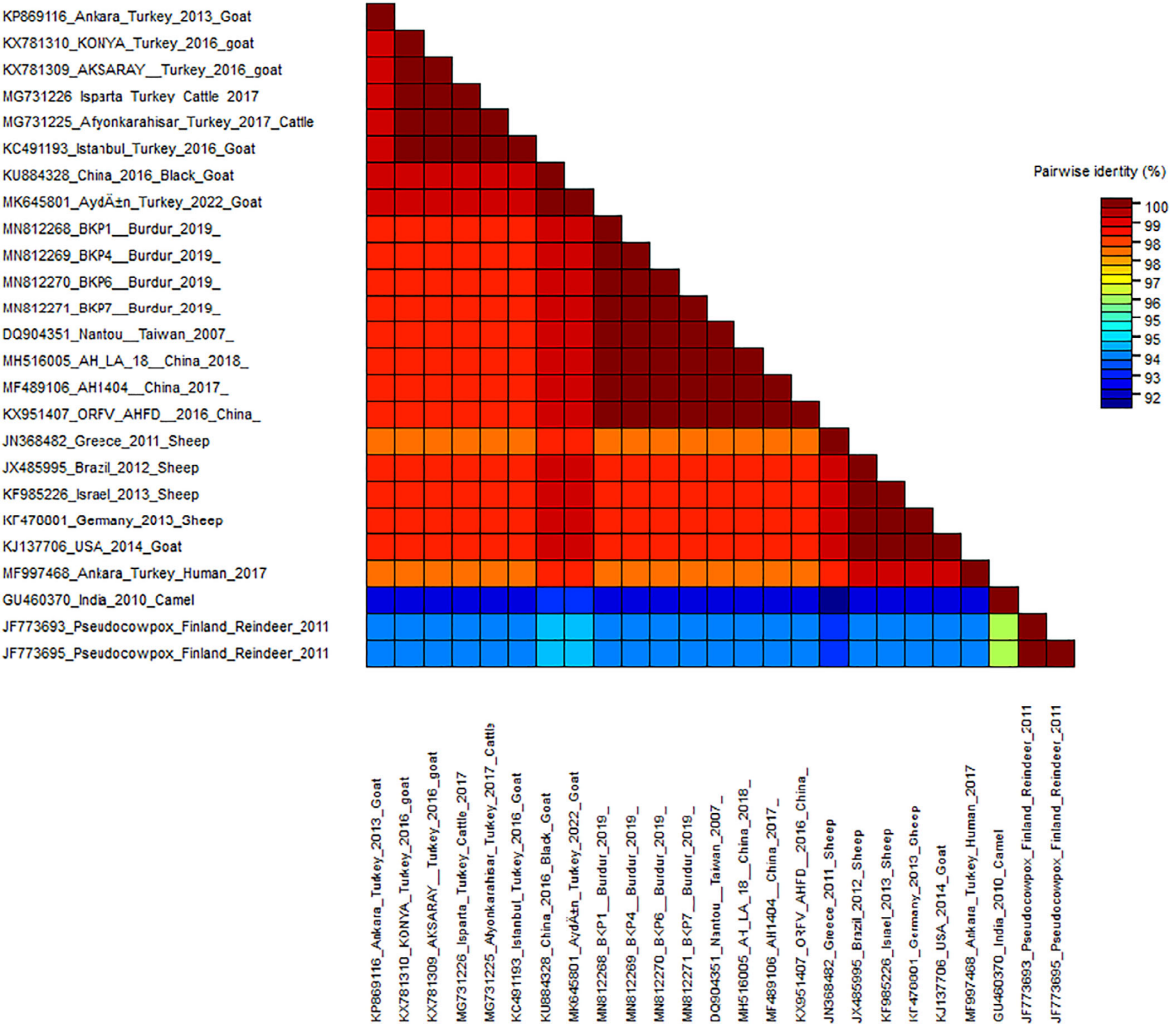
The sequence demarcation tool (SDT) results.

The sequence obtained using PP3 and PP4 oligonucleotide primers was subjected to phylogenetic analysis (Figure 1). Figure 1 shows the genetic relationship of the amplified sequence to sequences of reference strains from the GenBank. According to the phylogenetic analyses, it was seen that the virus in our samples was located in Group I. In this study, it was determined that the samples detected as positive showed 100% identity with the samples detected in the Chinese and Taiwan isolates. In addition, when the identity rates of the samples in this study were compared, it was determined that they were 100% identities (Figure 2). The GenBank accession numbers of the sequences are as follows: MN812268 (BKP1‐TR2019), MN812269 (BKP4‐TR2019), MN812270 (BKP6‐TR2019), MN812271 (BKP7‐TR2019).

The nucleotide sequences of our field isolates (BKP1, BKP4, BKP6 and BKP7) showed 100% similarity with orf strains detected from goats in China in 2016 (KX951407 ORFV/AH‐FD 2016/China), 2017 (MF489106 AH1404 China/2017), 2018 (MH516005 AH/LA/18 China /2018) and Taiwan in 2007 (DQ904351 Nantou Taiwan/2007). Conversely, it was determined that the sample exhibited a 98.8% similarity to ORFV strains identified in cattle from Afyonkarahisar and Isparta provinces in Türkiye in 2017 (GenBank accession numbers MG731225 for Afyonkarahisar/Türkiye 2017 Cattle and MG731226 for Isparta/Türkiye 2017 Cattle). In addition, the nucleotide similarity of our field strains obtained in our study with orf strains detected from goats in Ankara (Türkiye) in 2013 (KP869116 Ankara/Türkiye 2013 Goat), Aksaray and Konya (Türkiye) in 2016 (KX781309 Aksaray/Türkiye 2016 Goat; KX781310 Konya/Türkiye 2016 Goat) and Aydın in 2022 (MK645801 Aydın/Türkiye 2022 Goat) was determined as 98.8%. In addition, our isolates show 98.3% similarity to the human strain (MF997468) detected in Ankara/Türkiye in 2017. As a result of our research, it was determined that the strains we obtained were genetically similar (100%) among themselves based on a partial B2L gene. In the phylogenetic tree we constructed, all orf strains other than MF997468 (Ankara/Türkiye Human 2017 isolate) were detected from humans in Türkiye, and the strains we isolated were classified in Group I (Figure 2).

**FIGURE 3:**
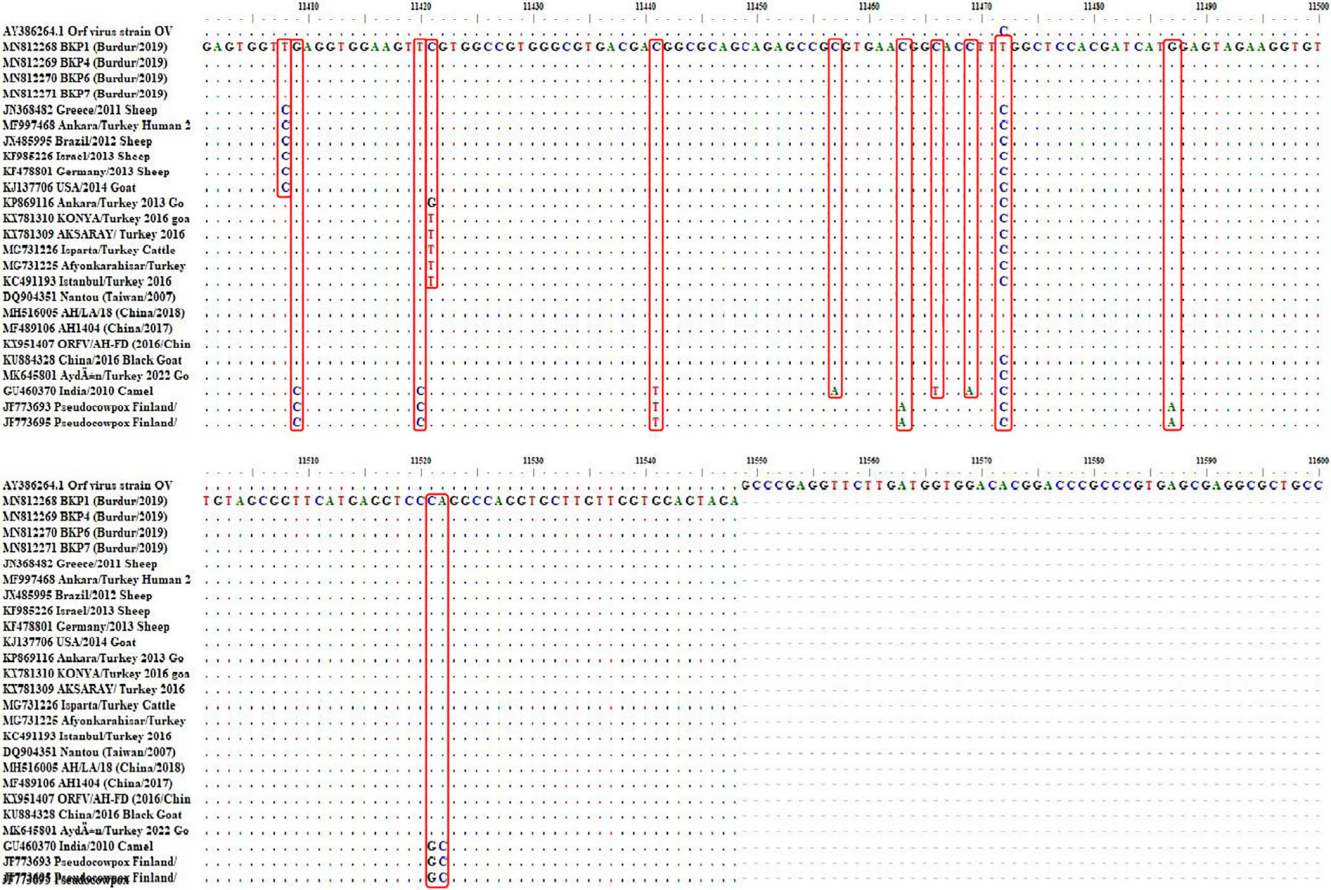
The nucleotide differences between orf virus isolates isolated in the study and accessed via GenBank.

To determine the nucleotide changes in the B2L genomic regions of the orf strains detected in our study, the genome sequences of ORFV strains (21 units) isolated from different species in Türkiye and around the world were compared with the genome sequences available in GenBank. The generated dataset was aligned using the MUSCLE program, and sequences in the same frame were manually checked for nucleotide changes using the AliView program. The ‘Thymine‐Cytosine’ mutations at positions 11,408 and 11,472 in the sequence account for the differentiation of two distinct groups observed in the phylogenetic tree (Figure 3).

No mastitis‐related changes were observed in the milk or mammary examinations. It was determined that the viral agent only caused localized skin lesions, resulting in partial difficulties during milking.

## 4 Discussion

ORFV causes dermatological infections in many animal species, mainly sheep and goats. The infection primarily manifests as pustular dermatitis in the oral cavity and labia, but can also occur in the genital area, foot and mammary area. The disease has a high morbidity rate, close to 100%, and is transmitted through contact and milk suckling between animals (Bergqvist, Kurban, and Abbas 2017; MacLachlan et al. 2017).

Consequently, molecular methods have replaced conventional laboratory techniques in the detection of ORFV, with semi‐nested PCR being a highly specific and rapid diagnostic method. Differentiation of ORFV from other parapoxvirus species is only possible through PCR and genomic characterization methods. In our study, we employed the semi‐nested PCR technique to diagnose ORFV in tissue samples collected from goats with dermatological lesions on their mammary tissue, and viral DNA was detected in four samples. ORFV is known to be transmitted iatrogenically during surgical treatments, through hand contact, and during ear tagging (Allworth et al. 1987). In our study, we hypothesized that the detection of lesions similar to ORFV infection on the mammary of all animals was due to hand milking and the farm workers' failure to adhere to hygiene and disinfection protocols (Akkutay‐Yoldar, Oguzoglu, and Akça 2016; Karakas et al. 2013; Şevik 2017; Şevik 2019).

In literature, molecular studies on ORFV have yet to establish a widely accepted formal genotyping system. Nonetheless, recent research has proposed informal genotyping approaches, often revealing three primary genotypes based on phylogenetic tree topologies. Although these classifications are not universally endorsed by authorities, they provide insight into the genetic diversity and evolutionary relationships of ORFV strains (Kumar et al. 2014; Koç 2022).

Countries have developed many prophylactic practices against contagious ecthyma. In Türkiye, attenuated live vaccines produced by originating Penorf CE strains are used for the protection and control of disease in sheep and goats.

The detected orf strains were found to be genetically close to strains isolated from studies conducted in Asian countries. However, the genetic similarity of the orf strains from Türkiye to those found in this study was lower compared to those from Asia. These findings suggest that while phylogenetic studies can offer general insights into the spread of viral strains, pinpointing the geographical origins of variants is more complex. In addition, the ORFV strains isolated from cattle in Türkiye were found to have a closer genetic relationship than those isolated from goats. This may be due to the virus's lack of species specificity. The co‐breeding of farm animals and collective care/feeding practices in the studied farms could be a factor in the transmission of pathogens across different species.

## 5 Conclusion

In conclusion, our research provided valuable data regarding the molecular epidemiology of ORFV variants among goats. Detecting local strains of the virus and understanding their genetic affinities with other strains can facilitate genotype‐specific vaccine production and inform future research techniques. Furthermore, further studies are needed to gain a better understanding of the circulation of ORFV between different species.

## Author Contributions

**Yakup Yıldırım**: writing–review and editing, writing–original draft, visualization; methodology, investigation, formal analysis, data curation, software. **Seval Bige Dağalp**: writing–review and editing, formal analysis, methodology, data curation, software. **Gökhan Bozkurt**: writing–review and editing, investigation, methodology. **Fırat Doğan, Ali Küçük**: writing–review and editing, investigation, methodology, data curation, software. All authors read and approved the manuscript.

## Ethics Statement

The Burdur Mehmet Akif Ersoy University Local Ethics Committee for Animal Experiments (MAKÜ‐HADYEK/2023‐1097), Burdur, Türkiye, gave its clearance before this study could be conducted.

## Conflicts of Interest

The authors declare no conflicts of interest.

### Peer Review

The peer review history for this article is available at https://publons.com/publon/10.1002/vms3.70139.

## Data Availability

The data that support the findings of this study are available from the corresponding author upon reasonable request.
